# Diagnostic Performance of a Mnemonic for Warning Symptoms in Predicting Acute Coronary Syndrome Diagnosis: A Retrospective Cross-Sectional Study

**DOI:** 10.3389/ijph.2023.1606115

**Published:** 2023-08-15

**Authors:** Attakowit Sattayaraksa, Thareerat Ananchaisarp, Polathep Vichitkunakorn, Ply Chichareon, Siriwimon Tantarattanapong

**Affiliations:** ^1^ Division of Family and Preventive Medicine, Prince of Songkla University, Songkhla, Thailand; ^2^ Cardiology Unit, Division of Internal Medicine, Prince of Songkla University, Songkhla, Thailand; ^3^ Division of Emergency Medicine, Prince of Songkla University, Songkhla, Thailand

**Keywords:** acronym, acute coronary syndrome, diagnostic performance, mnemonic, warning symptoms

## Abstract

**Objectives:** We aimed to create a mnemonic for acute coronary syndrome (ACS) warning symptoms and determine its diagnostic performance.

**Methods:** This retrospective cross-sectional study included patients visiting the emergency room with symptoms of suspected ACS during 2020–2021. The mnemonic was created using symptoms with an odds ratio (OR) for predicting ACS >1.0. The mnemonic with the highest OR and sensitivity was identified. Sensitivity analysis was performed to test the diagnostic performance of the mnemonic by patient subgroups commonly exhibiting atypical symptoms.

**Results:** ACS prevalence was 12.2% (415/3,400 patients). The mnemonic, “RUSH ChesT” [if you experience referred pain (R), unexplained sweating (U), shortness of breath (S), or heart fluttering (H) together with chest pain (C), visit the hospital in a timely (T) manner] had the best OR [7.81 (5.93–10.44)] and sensitivity [0.81 (0.77–0.85)]. This mnemonic had equal sensitivity in men and women, the elderly and adults, smokers and non-smokers, and those with and without diabetes or hypertension.

**Conclusion:** The “RUSH ChesT” mnemonic shows good diagnostic performance for patient suspected ACS. It may effectively help people memorize ACS warning symptoms.

## Introduction

Acute coronary syndrome (ACS) is a disease caused by blockage of the coronary artery supplying the heart muscles [1]. A severe myocardial infarction could be fatal [1]. According to the World Health Organization, ACS was the leading cause of death globally between 2000 and 2019 [2] and the third leading cause of death in the Thai population in 2019 [3]. Unstable angina (UA), non-ST-elevation myocardial infarction (NSTEMI), and ST-elevated myocardial infarction (STEMI) are the three subtypes of ACS, each with a different level of urgency for treatment [1, 4, 5]. For example, patients with STEMI should be treated by opening the coronary arteries within 3 h of symptom onset [4], whereas patients with NSTEMI or UA do not require urgent coronary intervention. However, all ACS subtypes exhibit similar symptoms [1, 4, 5]. Therefore, patients with suspected ACS symptoms should contact emergency medical services (EMS) for immediate hospital transfer. Owing to the fact that treatment time is critical, hospitals worldwide have developed STEMI fast-tracks to provide care to patients suspected with ACS. Many studies have revealed that the STEMI fast-track system significantly reduces the time of reperfusion therapy and mortality rates [6]; however, the mortality and morbidity rates of patients with ACS remain relatively high. One possible cause of this problem could be prolonged pre-hospital time (the time between the onset of ACS symptoms and arrival at the emergency room [ER]), which has been linked to increased mortality and morbidity rates in previous studies [7–10]. According to these studies, the mean pre-hospital time for patients with ACS in most countries ranged from 1.5 to 6 h [11–15].

A prolonged pre-hospital time may be caused by patients not realizing that their symptoms are those of ACS and that they should go to the hospital quickly, which is facilitated by calling EMS. Previous studies have revealed that knowing the warning symptoms of ACS and using EMS can significantly reduce pre-hospital time in patients with ACS [16, 17]. Generally, healthcare providers educate high-risk hospital patients about ACS through various advertisements and educational leaflets. Nevertheless, previous studies have found low knowledge about ACS symptoms [14] and low use of EMS [17]. In contrast, regarding cerebrovascular accidents (CVA), which also require urgent treatment, the “FAST” mnemonic [18] was invented to help the public remember the warning symptoms of CVA and promptly go to the hospital. It has been found that “FAST” mnemonics can help raise public awareness of CVA symptoms and EMS use, increasing the rate of thrombolytic therapy [19]. Nowadays, “FAST” mnemonics are used in many countries worldwide, and these have been modified to other mnemonics such as “BEFAST” in some countries [20]. Previous information on the mnemonics of ACS warning symptoms such as the “STOP” mnemonic (S - Sometimes wrong and symptoms can start slowly; T - Tightness or pain in the chest, pain in the arm, neck or jaw; O - Other symptoms such as shortness of breath, nausea or sweating; P - Phone 999 immediately) is inadequate. Furthermore, there is no report on their diagnostic performance, and mnemonics have not yet been used in an educational campaign. Therefore, this study aimed to create a mnemonic for the warning symptoms of ACS and determine its diagnostic performance.

## Methods

### Study Design and Setting

This retrospective cross-sectional study was conducted between 1 January 2020 and 31 December 2021. The study setting was the Songklanagarind hospital with a medical school, residency training programs, and a referral center.

### Study Sample and Sampling

We recruited all patients aged ≥18 years who visited the ER with suspected ACS symptoms (i.e., chest pain, epigastrium pain, dyspnea, sweating, palpitation, presyncope/syncope, nausea, dizziness, neck pain, jaw pain, or shoulder pain) between 1 January 2020 and 31 December 2021. We excluded patients with ACS who were referred from another hospital because this increased the prevalence of ACS and those who could not provide their medical history by themselves. Both the conditions may have influenced the diagnostic performance of ACS symptoms.

The sample size was calculated using an infinite population proportion formula: n_0_ = [(Z_1-α/2_)^2^P(1-P)]/d2 = 385. Further, no previous data were available; therefore, the prevalence was replaced with 0.50 to obtain the highest n_0_. Error (d) = 0.05, and alpha (α) = 0.05. We then calculated the required sample size as n = n_0_/prevalence = 1,406 and the prevalence of patients with ACS symptoms diagnosed with ACS = 0.274 [21]. Subsequently, the researcher calculated 30% for incomplete data as follows: *n* = 1,406/0.7 = 2009. Therefore, we reviewed the medical records of at least 2009 eligible participants.

### Variables

The independent variables were the baseline characteristics of participants and the presenting symptoms of each patient, which can be warning symptoms of ACS [4, 5] including chest pain, epigastric pain, dyspnea, sweating, palpitation, presyncope, syncope, nausea, dizziness, neck pain, jaw pain, and shoulder pain. For certain medical records that did not specify warning symptoms, we recoded these instances to “NA” as it was unclear whether this arose from participants not having symptoms or physician not asking about these symptoms.

The dependent variable in our study was the diagnosis of patients, defined as a “provisional diagnosis of ACS” in the medical record by an internal medicine physician or an emergency medicine physician (if the internal medicine consultation was not submitted). It was then categorized as UA, NSTEMI, STEMI, or non-ACS. Finally, we collected the final diagnosis from the discharge summary note for patients admitted Songklanagarind Hospital as a “definite diagnosis of ACS.”

### Data Collection

The hospital numbers of patients who were aged ≥18 years and underwent electrocardiography (ECG) in the ER were obtained from the Division of Digital Innovation and Data Analytics. Subsequently, all relevant medical records in the hospital information system were reviewed. Patients who presented with symptoms suspected of ACS were assigned an ID number and the relevant information was collected.

### Method of Creating the Mnemonic

To create the mnemonic, we first analyzed the diagnostic performance of each presenting symptom. Second, symptoms with an odds ratio (OR) >1.0 were selected to create the mnemonic. Third, following the ACS guidelines [4, 5], we created three mnemonics with chest pain as the primary symptom and other symptoms qualified in the previous step. Fourth, the diagnostic performance of each mnemonic was calculated in both the original and imputation datasets. Finally, all authors (emergency medicine doctor, family medicine doctors, and cardiologist) discussed the diagnostic performance of the three mnemonics and selected the most appropriate mnemonic to educate people in the future. We considered both high OR [a diagnostic performance that indicates the ability to predict disease probability well because it simultaneously considers both likelihood ratio positive (LR+) and LR−] and an acceptable specificity to avoid undue burden on the ER.

### Data Management and Analysis

The data were entered into Microsoft Excel 2019 and analyzed using R software version 4.2.2. Categorical data are presented as frequencies and percentages, while continuous data are presented as medians (Q1, Q3) when the normal distribution assumption was not met. Chi-square tests were used to examine the relationship between categorical variables between the ACS and non-ACS groups. The diagnostic performance of each symptom and mnemonic were calculated, consisting of 1) sensitivity (the probability of the presence of a symptom among patients with a confirmed diagnosis of ACS), 2) specificity (the probability of the absence of a symptom among non-ACS patients), 3) positive predictive value (PPV; the probability that a patient with a symptom result was actually diagnosed with ACS), 4) negative predictive value (NPV; the probability that a patient without a symptom result was not diagnosed with ACS), 5) likelihood ratio (LR; the number of times patients with each symptom presentation are more likely to be diagnosed of ACS than non-ACS), calculated as simple proportions using a 2 × 2 cross-tabulation of symptom (present or absent) versus diagnosis (ACS or non-ACS); and 6) OR (95% confidence interval [CI]), calculated using the logistic regression model. The statistical significance level was set at *p* < 0.05.

Missing data were managed with the multiple imputations method using the Amelia package to fill in missing values for each variable [22], and sensitivity analysis was performed to compare the diagnostic performance of each symptom and mnemonic between the imputation dataset (populated missing values) and the original dataset (containing missing values). Using the bootstrap method, we tested the internal validity of the diagnostic performance of the mnemonic. A stratified analysis was performed to evaluate the diagnostic performance of the mnemonic in patient subgroups commonly associated with atypical clinical presentation of ACS, as defined by the ESC guidelines [5] and previous studies [23, 24]. These subgroups were based on age, sex, obesity, smoking status, and underlying diabetes mellitus (DM) or hypertension.

## Results

We recruited 3,400 patients from the ER who presented symptoms suspected of ACS (1,546 patients in 2020 and 1854 in 2021); the provisional diagnosis was ACS in 415 patients (12.2%; 95% CI: 11.1–13.4). Table 1 shows the baseline characteristics of the participants divided based on diagnosis. We found that the proportion of males and the elderly (aged ≥60 years) was significantly higher in the ACS group than in the non-ACS group. In addition, smoking history was found to be significantly associated with ACS diagnosis. Finally, patients with a final diagnosis of ACS had a significantly higher proportion of comorbidities (except CVA) than those without ACS.

**TABLE 1:** Baseline characteristics divided based on diagnosis (*n* = 3,400) Thailand 2020–2021.

Characteristic	Total	Final diagnosis		
		ACS (*n* = 415)	Non-ACS (*n* = 2,985)	*p*-value^a^
Age group				<0.001
Age ≥60 years	2,069 (60.9)	296 (71.3)	1,773 (59.4)	
Age <60 years	1,331 (39.1)	119 (28.7)	1,212 (40.6)	
Sex				<0.001
Male	1734 (51.0)	311 (74.9)	1,423 (47.7)	
Female	1,666 (49.0)	104 (25.1)	1,562 (52.3)	
BMI				0.103
Obesity (BMI ≥25)	1,130 (40.8)	153 (41.4)	977 (40.7)	
Overweight (BMI 23–24.9)	603 (21.8)	94 (25.4)	509 (21.2)	
Normal (BMI 18.5–22.9)	856 (30.9)	107 (28.9)	749 (31.2)	
Underweight (BMI <18.5)	179 (6.5)	16 (4.3)	163 (6.8)	
History of smoking^b^				<0.001
Current smoker	210 (10.2)	65 (22.8)	145 (8.2)	
Ex-smoker	215 (10.4)	36 (12.6)	179 (10.1)	
Never smoked	1,633 (79.3)	184 (64.6)	1,449 (81.7)	
Underlying disease				
Hypertension	1,502 (44.2)	228 (54.9)	1,274 (42.7)	<0.001
Dyslipidemia	1,198 (35.2)	173 (41.7)	1,025 (34.3)	0.003
Coronary artery disease	838 (24.6)	190 (45.8)	648 (21.7)	<0.001
Diabetes mellitus	799 (23.5)	147 (35.4)	652 (21.8)	<0.001
Cerebrovascular accident	259 (7.6)	32 (7.7)	227 (7.6)	0.939
Peripheral arterial disease	32 (0.9)	9 (2.2)	23 (0.8)	0.006

^a^ Chi-square test.

^b^ History of smoking [27]: never smoked (patient had no previous history of smoking), ex-smoker (patient was able to quit smoking for ≥3 months), current smoker (patient is currently smoking or has quit for <3 months).

Data are presented as n (%). Abbreviations: ACS, acute coronary syndrome; BMI, body mass index.

Table 2 shows the presenting symptoms of the participants divided based on the final diagnosis. Patients with chest pain, referred pain, sweating, dyspnea, or palpitations were diagnosed with ACS at a significantly higher rate than those without these symptoms. In contrast, patients without presyncope symptoms had a higher proportion of ACS diagnoses than patients with presyncope symptoms.

**TABLE 2:** Presenting symptoms divided based on diagnosis. Thailand 2020–2021.

Presenting symptoms	Total*	Diagnosis**		
		ACS	Non-ACS	*p*-value^a^
Chest pain (*n* = 2,995)				<0.001
Chest pain	1,906 (63.6)	367 (19.3)	1,539 (80.7)	
No chest pain	1,089 (36.4)	34 (3.1)	1,055 (96.9)	
Referred pain*** (*n* = 1,865)				<0.001
Referred pain	472 (25.3)	139 (29.4)	333 (70.6)	
No referred pain	1,393 (74.7)	160 (11.5)	1,233 (88.5)	
Sweating (*n* = 1,969)				<0.001
Sweating	452 (23.0)	132 (29.2)	320 (70.8)	
No sweating	1,517 (77.0)	180 (11.9)	1,337 (88.1)	
Dyspnea (*n* = 2,182)				0.009
Dyspnea	909 (41.7)	140 (15.4)	769 (84.6)	
No dyspnea	1,273 (58.3)	146 (11.5)	1,127 (88.5)	
Palpitation (*n* = 2,535)				0.026
Palpitation	779 (30.7)	116 (14.9)	663 (85.1)	
No palpitation	1,756 (69.3)	204 (11.6)	1,552 (88.4)	
Nausea (*n* = 1,356)				0.677
Nausea	424 (31.3)	28 (6.6)	396 (93.4)	
No nausea	932 (68.3)	69 (7.4)	863 (92.6)	
Dizziness (*n* = 497)				0.953
Dizziness	390 (78.5)	19 (4.9)	371 (95.1)	
No dizziness	107 (21.5)	6 (5.6)	101 (94.4)	
Presyncope (*n* = 1,196)				0.019
Presyncope	379 (31.7)	32 (8.4)	347 (91.6)	
No presyncope	817 (68.3)	109 (13.3)	708 (86.7)	
Epigastrium pain (*n* = 875)				0.069
Epigastrium pain	764 (87.3)	39 (5.1)	725 (94.9)	
No epigastrium pain	111 (12.7)	11 (9.9)	100 (90.1)	
Syncope (*n* = 670)				0.115
Syncope	71 (10.6)	3 (4.2)	68 (95.8)	
No syncope	599 (89.4)	66 (11.0)	533 (89.0)	

^a^ Chi-square test.

*Column percent.

**Row percent.

***Referred pain: Neck, shoulder, or jaw pain.

Data are presented as n (%). Abbreviations: ACS, acute coronary syndrome.

Table 3 shows the diagnostic performance of each presenting symptom suspected of presenting with ACS, compared between the imputation dataset and the original dataset. “Chest pain” had the highest ORs and sensitivity for ACS diagnosis [OR (95% CI) = 7.40 (5.24–10.79); sensitivity (95% CI) = 0.92 (0.88–0.94)]. The other symptoms with an OR > 1.0 were referred pain [OR (95% CI) = 3.22 (2.49–4.16)], sweating [OR (95% CI) = 3.06 (2.37–3.95)], dyspnea [OR (95% CI) = 1.41 (1.10–1.80)], and palpitation [OR (95% CI) = 1.33 (1.04–1.70)]. The ORs of referred pain and sweating in the imputation dataset [ORs (95% CI) = 1.50 (1.22, 1.84) and 1.88 (1.51, 2.32), respectively] were significantly reduced compared to the original dataset [ORs (95% CI) = 3.22 (2.49, 4.16) and 3.06 (2.37, 3.95), respectively]. Table 4 shows the diagnostic performance of the three mnemonics based on the original and imputation datasets. Each mnemonic in the imputation dataset performed similarly to that of the original dataset. The first, which included chest pain and one additional symptom in “dyspnea or sweating or palpitation or referred pain,” was selected by three specialists as having the best diagnostic performance for ACS [OR (95% CI) = 7.81 (5.93–10.44); sensitivity (95% CI) = 0.81 (0.77–0.85)] and had acceptable specificity. This mnemonic was created to make it easy for patients to remember “RUSH ChesT,” which means “if you have referred pain (R), unexplained sweating (U), shortness of breath (S), or heart fluttering (H) together with chest pain, you should go to hospital Timely (T).”

**TABLE 3:** Diagnostic performance of each symptom for ACS diagnosis: a sensitivity analysis using original and imputation datasets. Thailand 2020–2021.

Symptoms	Odds ratio (95% CI)	Sensitivity (95% CI)	Specificity (95% CI)	PPV (95% CI)	NPV (95% CI)	LR+ (95% CI)	LR- (95% CI)
Chest pain							
Original dataset (*n* = 2,995)	7.40 (5.24, 10.79)	0.92 (0.88, 0.94)	0.41 (0.39, 0.43)	0.19 (0.18, 0.21)	0.97 (0.96, 0.98)	1.54 (1.48, 1.61)	0.21 (0.15, 0.29)
Imputation dataset	6.48 (4.72, 9.15)	0.90 (0.87, 0.93)	0.42 (0.40, 0.43)	0.18 (0.16, 0.19)	0.97 (0.96, 0.98)	1.54 (1.48, 1.61)	0.24 (0.18, 0.32)
Referred pain^a^							
Original dataset (*n* = 1,865)	3.22 (2.49, 4.16)	0.46 (0.41, 0.52)	0.79 (0.77, 0.81)	0.29 (0.25, 0.34)	0.89 (0.87, 0.90)	2.19 (1.87, 2.55)	0.68 (0.61, 0.76)
Imputation dataset	1.50 (1.22, 1.84)	0.50 (0.45, 0.55)	0.60 (0.59, 0.62)	0.15 (0.13, 0.17)	0.90 (0.88, 0.91)	1.25 (1.12, 1.39)	0.84 (0.76, 0.92)
Sweating							
Original dataset (*n* = 1,969)	3.06 (2.37, 3.95)	0.42 (0.37, 0.48)	0.81 (0.79, 0.83)	0.29 (0.25, 0.34)	0.88 (0.86, 0.90)	2.19 (1.86, 2.58)	0.72 (0.65, 0.79)
Imputation dataset	1.88 (1.51, 2.32)	0.39 (0.34, 0.44)	0.75 (0.73, 0.76)	0.18 (0.15, 0.20)	0.90 (0.89, 0.91)	1.54 (1.34, 1.76)	0.82 (0.75, 0.89)
Dyspnea							
Original dataset (*n* = 2,182)	1.41 (1.10, 1.80)	0.49 (0.43, 0.55)	0.59 (0.57, 0.62)	0.15 (0.13, 0.18)	0.89 (0.87, 0.90)	1.21 (1.06, 1.37)	0.86 (0.76, 0.97)
Imputation dataset	1.13 (0.921.39)	0.44 (0.39, 0.49)	0.59 (0.57, 0.61)	0.13 (0.11, 0.15)	0.88 (0.87, 0.90)	1.07 (0.96, 1.21)	0.95 (0.87, 1.04)
Palpitation							
Original dataset (*n* = 2,535)	1.33 (1.04, 1.70)	0.36 (0.31, 0.42)	0.70 (0.68, 0.72)	0.15 (0.12, 0.18)	0.88 (0.87, 0.90)	1.21 (1.03, 1.42)	0.91 (0.83, 0.99)
Imputation dataset	1.30 (1.05, 1.61)	0.37 (0.33, 0.42)	0.69 (0.67, 0.70)	0.14 (0.12, 0.16)	0.89 (0.87, 0.90)	1.19 (1.04, 1.36)	0.91 (0.85, 0.99)
Nausea							
Original dataset (*n* = 1,356)	0.88 (0.55, 1.38)	0.29 (0.20, 0.39)	0.69 (0.66, 0.71)	0.07 (0.04, 0.09)	0.93 (0.91, 0.94)	0.92 (0.66, 1.27)	1.04 (0.91, 1.18)
Imputation dataset	1.03 (0.83, 1.27)	0.39 (0.35, 0.44)	0.61 (0.60, 0.63)	0.12 (0.11, 0.14)	0.88 (0.86, 0.89)	1.02 (0.90, 1.16)	0.99 (0.91, 1.07)
Dizziness							
Original dataset (*n* = 497)	0.86 (0.35, 2.42)	0.76 (0.55, 0.91)	0.21 (0.18, 0.25)	0.05 (0.03, 0.08)	0.94 (0.88, 0.98)	0.97 (0.77, 1.21)	1.12 (0.55, 2.30)
Imputation dataset	0.97 (0.78, 1.23)	0.72 (0.67, 0.76)	0.27 (0.26, 0.29)	0.12 (0.11, 0.13)	0.88 (0.85, 0.90)	0.99 (0.93, 1.06)	1.02 (0.86, 1.20)
Presyncope							
Original dataset (*n* = 1,196)	0.60 (0.39, 0.90)	0.23 (0.16, 0.31)	0.67 (0.64, 0.70)	0.08 (0.06, 0.12)	0.87 (0.84, 0.89)	0.69 (0.50, 0.95)	1.15 (1.04, 1.27)
Imputation dataset	1.01 (0.81, 1.25)	0.35 (0.31, 0.40)	0.65 (0.63, 0.66)	0.12 (0.10, 0.14)	0.88 (0.86, 0.89)	1.01 (0.87, 1.15)	1.00 (0.92, 1.08)
Epigastrium pain							
Original dataset (*n* = 875)	0.49 (0.25, 1.03)	0.78 (0.64, 0.88)	0.12 (0.10, 0.15)	0.05 (0.04, 0.07)	0.90 (0.83, 0.95)	0.89 (0.76, 1.03)	1.81 (1.04, 3.16)
Imputation dataset	0.77 (0.60, 0.99)	0.78 (0.74, 0.82)	0.18 (0.17, 0.19)	0.12 (0.10, 0.13)	0.85 (0.82, 0.88)	0.95 (0.90, 1.00)	1.23 (1.01, 1.50)
Syncope							
Original dataset (*n* = 670)	0.36 (0.09, 0.99)	0.04 (0.01, 0.12)	0.89 (0.86, 0.91)	0.04 (0.01, 0.12)	0.89 (0.86, 0.91)	0.38 (0.12, 1.19)	1.08 (1.02, 1.14)
Imputation dataset	0.76 (0.55, 1.03)	0.12 (0.09, 0.16)	0.85 (0.83, 0.86)	0.10 (0.07, 0.13)	0.87 (0.86, 0.89)	0.79 (0.60, 1.03)	1.04 (1.00, 1.08)

^a^ Referred pain: neck, shoulder, or jaw pain.

Abbreviations: CI, confidence interval; PPV, positive predictive value; NPV, negative predictive value; LR+, positive likelihood ratio; LR-, negative likelihood ratio.

**TABLE 4:** Diagnostic performance of three mnemonics for ACS diagnosis: a sensitivity analysis based on original and imputation datasets. Thailand 2020–2021.

Mnemonics	Odds ratio (95% CI)	Sensitivity (95% CI)	Specificity (95% CI)	PPV (95% CI)	NPV (95% CI)	LR+ (95% CI)	LR- (95% CI)
Chest pain and “dyspnea or sweating or palpitation or referred pain^a^”							
Original dataset	7.81 (5.93, 10.44)	0.81 (0.77, 0.85)	0.64 (0.62, 0.66)	0.28 (0.25, 0.30)	0.95 (0.94, 0.96)	2.28 (2.11, 2.46)	0.29 (0.23, 0.36)
Imputation dataset	4.75 (3.76–6.05)	0.76 (0.71, 0.80)	0.60 (0.58, 0.62)	0.21 (0.19, 0.23)	0.95 (0.94, 0.96)	1.90 (1.78, 2.04)	0.40 (0.34, 0.48)
Chest pain and “dyspnea or palpitation or referred pain^a^”							
Original dataset	6.71 (5.15, 8.83)	0.77 (0.72, 0.81)	0.67 (0.65, 0.69)	0.27 (0.24, 0.30)	0.95 (0.94, 0.96)	2.33 (2.14, 2.53)	0.35 (0.28, 0.42)
Imputation dataset	4.09 (3.28–5.14)	0.71 (0.66, 0.75)	0.62 (0.61, 0.64)	0.21 (0.19, 0.23)	0.94 (0.93, 0.95)	1.89 (1.75, 2.05)	0.46 (0.40, 0.54)
Chest pain and “sweating or palpitation or referred pain^a^”							
Original dataset	6.54 (5.07, 8.50)	0.71 (0.66, 0.76)	0.73 (0.71, 0.75)	0.28 (0.25, 0.31)	0.94 (0.93, 0.95)	2.60 (2.36, 2.87)	0.40 (0.33, 0.47)
Imputation dataset	4.24 (3.41–5.30)	0.68 (0.63, 0.73)	0.66 (0.65, 0.68)	0.22 (0.20, 0.24)	0.94 (0.93, 0.95)	2.03 (1.87, 2.21)	0.48 (0.41, 0.55)

Abbreviations: ACS, acute coronary syndrome; PPV, positive predictive value; NPV, negative predictive value; LR+, positive likelihood ratio; LR-, negative likelihood ratio.

^a^ Referred pain: neck, shoulder, or jaw pain.

Table 5 shows the internal validity of the “RUSH ChesT” mnemonic based on the bootstrap method. We found that the diagnostic performance of the bootstrap validation dataset did not differ from that of the original dataset.

**TABLE 5:** “RUSH ChesT” mnemonic diagnostic performance compared between the bootstrap validation dataset and complete dataset results. Thailand 2020–2021.

	Odds ratio (95% CI)	Sensitivity (95% CI)	Specificity (95% CI)	PPV (95% CI)	NPV (95% CI)	LR+ (95% CI)	LR- (95% CI)
Original dataset	7.81 (5.93, 10.44)	0.81 (0.77, 0.85)	0.64 (0.62, 0.66)	0.28 (0.25, 0.30)	0.95 (0.94, 0.96)	2.28 (2.11, 2.46)	0.29 (0.23, 0.36)
Bootstrap validation dataset	7.95 (5.38, 9.98)	0.81 (0.77, 0.85)	0.64 (0.62, 0.66)	0.28 (0.25, 0.30)	0.95 (0.94, 0.96)	2.28 (2.10, 2.46)	0.29 (0.23, 0.36)

Abbreviations: CI, confidence interval; PPV, positive predictive value; NPV, negative predictive value; LR+, positive likelihood ratio; LR-, negative likelihood ratio.

Table 6 shows the diagnostic performance of the “RUSH ChesT” mnemonic in the subgroup of patients who may present with atypical presentation. We found that the “RUSH ChesT” mnemonic had no statistically significant difference in OR and sensitivity between “male and female,” “elderly and adult,” “currently smoking and never/former smoking,” “obese and non-obese,” “with and without underlying DM,” and “with and without underlying hypertension” groups. In contrast, this mnemonic had higher specificity in the elderly individuals [0.68 (95% CI = 0.65–0.70)] and patients with underlying DM [0.71 (95% CI = 0.67–0.75)]. Moreover, this mnemonic had a higher positive predictive value in patients who currently smoked [0.55 (0.44, 0.65)] or had underlying hypertension [0.34 (0.30, 0.39)].

**TABLE 6:** Diagnostic performance of the “RUSH ChesT” mnemonic: a stratified analysis. Thailand 2020–2021.

	Odds ratio (95% CI)	Sensitivity (95% CI)	Specificity (95% CI)	PPV (95% CI)	NPV (95% CI)	LR+ (95% CI)	LR- (95% CI)
Sex							
Male	8.51 (6.09, 12.15)	0.83 (0.78, 0.87)	0.63 (0.60, 0.66)	0.38 (0.34, 0.42)	0.93 (0.91, 0.95)	2.26 (2.05, 2.49)	0.27 (0.20, 0.35)
Female	5.61 (3.42, 9.55)	0.75 (0.64, 0.84)	0.66 (0.63, 0.68)	0.14 (0.11, 0.18)	0.97 (0.96, 0.98)	2.17 (1.86, 2.51)	0.39 (0.27, 0.56)
Age groups							
<60 years	6.80 (4.11, 11.87)	0.82 (0.74, 0.89)	0.59 (0.56, 0.63)	0.20 (0.16, 0.24)	0.96 (0.94, 0.98)	2.02 (1.79, 2.29)	0.30 (0.19, 0.45)
≥60 years	8.79 (6.34, 12.43)	0.81 (0.75, 0.85)	0.68 (0.65, 0.70)	0.33 (0.29, 0.37)	0.95 (0.93, 0.96)	2.50 (2.26, 2.77)	0.28 (0.22, 0.37)
Smoking status							
Current smoker	6.83 (3.14, 16.24)	0.85 (0.74, 0.93)	0.54 (0.43, 0.64)	0.55 (0.44, 0.65)	0.85 (0.73, 0.93)	1.85 (1.45, 2.35)	0.27 (0.14, 0.51)
Never/former smoker	7.55 (5.16, 11.35)	0.81 (0.74, 0.86)	0.64 (0.61, 0.67)	0.26 (0.22, 0.30)	0.96 (0.94, 0.97)	2.25 (2.03, 2.50)	0.30 (0.22, 0.40)
Obesity							
Yes	10.36 (6.37, 17.74)	0.86 (0.79, 0.91)	0.63 (0.60, 0.67)	0.31 (0.26, 0.36)	0.96 (0.94, 0.98)	2.34 (2.07, 2.63)	0.23 (0.15, 0.34)
No	6.50 (4.52, 9.55)	0.78 (0.72, 0.84)	0.64 (0.61, 0.67)	0.29 (0.25, 0.33)	0.94 (0.92, 0.96)	2.20 (1.96, 2.46)	0.34 (0.26, 0.45)
Having underlying diabetes mellitus							
Yes	10.23 (6.38, 17.00)	0.81 (0.73, 0.87)	0.71 (0.67, 0.75)	0.42 (0.36, 0.49)	0.93 (0.90, 0.96)	2.79 (2.36, 3.29)	0.27 (0.19, 0.39)
No	7.34 (5.22, 10.53)	0.81 (0.76, 0.86)	0.62 (0.60, 0.65)	0.23 (0.20, 0.26)	0.96 (0.95, 0.97)	2.17 (1.99, 2.37)	0.30 (0.22, 0.39)
Having underlying hypertension							
Yes	9.16 (6.30, 13.66)	0.82 (0.76, 0.87)	0.67 (0.64, 0.70)	0.34 (0.30, 0.39)	0.95 (0.93, 0.96)	2.49 (2.23, 2.79)	0.27 (0.20, 0.37)
No	6.78 (4.53, 10.45)	0.81 (0.73, 0.86)	0.62 (0.59, 0.65)	0.22 (0.19, 0.26)	0.96 (0.94, 0.97)	2.13 (1.91, 2.37)	0.31 (0.23, 0.43)

Abbreviations: CI, confidence interval; PPV, positive predictive value; NPV, negative predictive value; LR+, positive likelihood ratio; LR-, negative likelihood ratio.

## Discussion

The “RUSH ChesT” mnemonic has a good diagnostic performance for educating people that if they have the symptoms included in the mnemonic, there is a high chance of having ACS, and that they should immediately contact EMS. We found that the mnemonic has high internal validity and is effective in females, older adults, patients with obesity, smokers, and patients with DM or hypertension who frequently present with atypical symptoms.

Compared to that observed in previous studies, the prevalence of ACS was lower in our study [25]. This could be because our study was retrospective and included all patients with suspected ACS who were selected from patients undergoing ECG and having any of the presenting symptoms of ACS. In contrast, the previous study was prospective and could only recruit patients with suspected ACS on the day of visiting the emergency department. In addition, a previous study [25] excluded patients with heart failure presenting with similar symptoms as those with ACS. However, we did not exclude this group from our study because we also considered heart failure as an emergency condition and that it is difficult for people to distinguish between these conditions. In this study, regarding the participants’ baseline characteristics, the median age of our participants was in the elderly group and most participants were obese, which is consistent with a previous study [25]. However, the proportions of male and female patients in our study were equal, whereas a previous study [25] included more males than females.

Patients with ACS exhibit multiple manifestations [4, 5]. We found that chest pain has the best OR and sensitivity as a symptom for ACS diagnosis, which corresponds to symptom data from patients with ACS in the ESC guideline 2020 [5] and Thai ACS guideline 2020 [4], which state that chest pain is the main symptom of patients with ACS. The sensitivity and specificity of chest pain in our study were better than those of a previous study [25], presumably because the previous study used the 13-Item ACS Checklist, which classified symptoms as chest pain, chest pressure, or chest discomfort. In contrast, in our study, these symptoms were classified as chest pain. The overall number of cases was averaged for each sub-symptom due to this separation, reducing the likelihood of obtaining information about patients who presented with the symptoms among patients with a confirmed diagnosis of ACS and did not present with the symptoms among non-ACS patients. The sensitivity and specificity of dyspnea, sweating, and palpitations were comparable to those in a previous study [25]. In screening patients with suspected ACS, the symptoms of nausea, syncope, and presyncope had relatively low OR and sensitivity values, consistent with previous findings [25], indicating that these were atypical ACS symptoms. Epigastric pain and dizziness are highly sensitive in screening for ACS; however, due to high missing data for these two symptoms, this result may be overestimated.

This mnemonic system may be useful for memorizing information regarding ACS warning symptoms. The results of the sensitivity and specificity of the “RUSH ChesT” mnemonic for ACS symptoms are comparable to those of the “FAST” mnemonic for stroke symptoms (sensitivity = 85.0% and specificity = 68.0%) [26]. Owing to the fact that this mnemonic mainly consisted of symptoms of the typical presentation of ACS, the subgroup analysis in our study revealed the same OR and sensitivity in females, people with obesity, current smokers, older adults, and patients with DM or hypertension, who may present with an atypical presentation, according to ESC guidelines [5] and previous studies [23, 24]. Further, because we aimed to help people memorize ACS warning symptoms and immediately visit the hospital to confirm the diagnosis, we created the mnemonic based on OR and sensitivity. The low specificity of this mnemonic was acceptable to emergency medicine doctor and cardiologist, and an ER triage system could compensate for it.

The strengths of this study are as follows: First, it is one of the few studies that created and tested mnemonics of ACS warning symptoms. Second, our study included data from all patients who visited the ER with symptoms suspected of ACS during a 2-year period, reducing the problem of selection bias. Third, the total number of patients in our study exceeded that reported in a previous study [25]. Moreover, patient data were received from the Hospital Information Systems which can help avoid data loss and reduce the risk of misinterpretation from the handwriting of the physician. This improves data collection accuracy. Finally, using bootstrap validation, the mnemonic scale was tested for internal validity.

This study had some limitations, the most notable of which being its retrospective design. First, the completeness of the data depends on the resolution of the medical record of the physician, which is difficult to achieve due to the emergency conditions of the patients. Therefore, according to the guidelines, most records in this study did not mention all symptoms that could be the presenting symptom of ACS [4, 5]. For example, epigastric pain was mentioned in only 26.0% of cases, syncope in only 20.0% of cases, and dizziness in only 15.0% of cases. Multiple missing data points for each presentation may influence the diagnostic performance for each symptom. We attempted to manage the missing data using the multiple imputation method. Although the ORs of referred pain and sweating in the imputation dataset decreased compared to the original dataset, they remained greater than 1.0, and as such, they were both incorporated into the mnemonic. For diagnostic performance of mnemonic, the results were identical between imputation dataset and complete dataset; therefore, we presented a complete dataset. Second, for the subgroup analysis, our analysis lacked data on some patient factors that have been shown in the ESC guidelines [5] to be associated with atypical presentations of ACS. This affects the diagnostic performance of the mnemonic within each group, such as in patients with chronic kidney disease and dementia. Third, we used weight data from records within the previous 3 months in some patients because weight data were not recorded on the day of the ER visit. This may have resulted in minor differences in the true patient weight. Fourth, due to the limitations of the retrospective study design, we used the provisional diagnosis for analysis rather than the definite diagnosis, which was a reference diagnosis of ACS. However, we analyzed the distinction between the provisional and definite diagnostic data. Only 3.95% of patients were provisionally diagnosed with ACS and subsequently changed to non-ACS at the final diagnosis (consisting of congestive heart failure, Brugada syndrome, severe aortic stenosis, pulmonary embolism, chronic obstructive pulmonary disease with acute exacerbation, and pericarditis). According to our findings, this inconsistency is not clinically significant because these are emergency conditions that require patients to consult a doctor promptly. Moreover, to use this mnemonic, the patient must have chest pain as the primary symptom, along with other symptoms listed in the mnemonic, according to the guidelines [4, 5]. Owing to the fact that each suspected ACS symptom can manifest in many other diseases besides ACS, this can help avoid overcrowding the ER. However, some patients with ACS (3.1% in our study) did not have chest pain as a presenting symptom, and a previous study found that 8.4% of patients with ACS in 14 countries presented without chest pain [23]. Therefore, when using this mnemonic in education, healthcare providers should be aware that patients with ACS who do not present with chest pain may be misdiagnosed. In addition, because this study was conducted in the ER of a tertiary care hospital, the findings may not apply to other hospital settings. Finally, excluding patients who were not able to independently report which ACS warning symptoms they experienced, such as patients whose relatives had to explain their symptoms, may have led to a decreased prevalence of ACS in this study, affecting the diagnostic performance of each symptom and the mnemonic.

To use this mnemonic in educating the public, it should be translated into the local language so that people can understand and remember it better. We recommend that the following future studies be conducted. First, it should be tested in other settings to determine the external validity of the mnemonic. Second, research involving participants is required to evaluate the effectiveness of using our mnemonic in helping patients to recognize ACS symptoms in real life. Finally, a prospective study using a standardized record listing of all symptoms suspected of ACS for use in the ER when evaluating patients suspected of having ACS may help physicians select relevant participants, reduce missing data for each presenting symptom, and determine a final diagnosis for all patients.

### Conclusion

The “RUSH ChesT” mnemonic has effective diagnostic performance for diagnosing ACS, including in women, patients with obesity, smokers, elderly patients, and patients with diabetes or hypertension. Therefore, this may be a valuable tool to use in public healthcare to educate people regarding warning symptoms of ACS.

## Abbreviations

ACS, cute coronary syndrome; OR, odds ratio; UA, unstable angina; NSTEMI, non-ST-elevation myocardial infarction; STEMI, ST elevated myocardial infarction; ER, emergency room; EMS, emergency medical services; CVA, cerebrovascular accident; PSU, Prince of Songkla University; PPV, positive predictive value; NPV, Negative predictive value; DM, diabetes mellitus; CI, confidence interval.
